# Observations on Preparation, Discrimination, Prevention, Reparation, Association, and Publication as Applied to Dentistry

**Published:** 1890-10

**Authors:** Charles B. Atkinson


### OBSERVATIONS ON PREPARATION, DISCRIMINATION, PREVENTION, REPARATION, ASSOCIATION, AND PUBLICATION AS APPLIED TO DENTISTRY.[1]

BY CHARLES B. ATKINSON, D.D.S.

PREPARATION includes selection as a first postulate, and directs attention to a consideration of the qualities essential and desirable for a dentist's success.[2]

Probably gentleness in handling the patient does more to increase a practice than any other single quality. Cleanliness of person and office are important elements. These, with courteous manners, have built more practices than the higher and more useful possessions of knowledge and skill.

Nevertheless patients respect a firm stand taken on a principle of practice, and indeed expect of the operator a directive force of character, to which they readily submit.

Clear and prompt diagnosis is not a quality given to all of us, nor can it be altogether acquired. Some of his qualifications the dentist must have born in him.

Comprehension, judgment, decision, firmness, ingenuity, persistence, neatness, patience, honesty, and modesty are qualities that can be fostered, but the bases of which must be bred in the man to effectively develop.

Education, manipulation, conversation are directly resultant on organized effort, and more immediately come under the possibility of acquisition.

The inbred qualities are especially those which control the possibility of possession of diagnostic ability.

Routine following of formulated aspects of disease does not constitute diagnosis. For which are required comprehension, perspicacity, and judgment pre-eminently.

Education necessarily attends these as taking partly the place of experience. Proper education would take the place of experience, as it should include in its scope not only extended clinical teaching, which of itself is experience, but a large range of cases in practice.

---

[1] Read before the New York Odontological Society, May, 1890.

[2] Well considered by Dr. Dean in *Dental Cosmos*, November and December, 1889.

Manual training is important to a great degree. Delicacy of manipulation is required so constantly in proper dental operations that the schooling of the hands can hardly be pursued too far.

The long-continued routine drawing in an artist's training tells when he comes to use the brush in his professional work; so also the years of exercises required of a pianist lay the habit of accuracy and delicacy of touch and control over the hands without which execution would be impossible. Dr. Barrett[1] has well considered this subject of manual training, but it is too vastly important not to be urged again and again.

Previous private pupilage is of great importance to a proper candidate for dental education.

Not all, by any means, of those who undertake the study of the profession are possessed of the proper inborn capacity, mental and physical, that are absolutely necessary to the only success to which a professional man should aspire. General fundamental education and technical training of the hands and eyes should come first, and pupilage follow, and it is here the weeding of improper material should come.

An uncouth, slovenly, immoral pupil, when proved to be incorrigible, should be dropped at once, as lacking some qualities necessary, and possessing others inimical, to continuing the study of dentistry.

Thus would material of proper quality be presented to the colleges for the necessary drill on text-book matter, and the more extended clinical opportunities of the infirmary, when has been reached a point of preparation of the moral, manual, and mental possibilities that invites rapid development to a degree of professional capacity very seldom reached under existing plans of preparation.

College education would advance to meet the demand, and American dentistry would again occupy the position before the world to which it has a right, but which it is threatened it will lose because of the indifference of the profession to the admission of unworthy members. The world has taken cognizance of our slack methods, and we have been aroused to a contemplation of what can be done to meet the issues before us. The enactment of laws governing the incoming operators has been secured, meeting more or less this phase of preparation.

These are steps to an end, all of them, and the end should be

---

[1] *Western Dental Journal*, May, 1889, "The Master and his Servants, also recently presented by Dr. Kirk before the First District Dental Society.

pushed higher and higher as our appreciation of the possibilities before us extends.

There are three especially weak points in most college curriculæ,—

Chemistry (underlying as it does all adequate comprehension of tissual alteration, and also of the medicines and materials we use) should constitute a first, broad, intimate, and intelligently-directed course. The first year devoted solely to chemistry and anatomy, didactic and practical, would be immensely beneficial to the student throughout his further course, both undergraduate and practising.

Diagnosis seems to be treated almost entirely as dependent on apparent surface indications. But, then, do not the majority of dentists consider extraction, the supply of crowns or teeth on plates, plastic and gold filling to embrace the practice of dentistry? This is perhaps natural, for what does the student notice?—a patient presents in the infirmary, the superintending demonstrator consults his pocket and locates and determines certain work to be done, —filling, crown, or plate,—and the patient is turned over to the student to carry out the programme. What is the result? The student's mind is at once directed to the mechanical aspects of the case, and his interest concentrated thereon. He is not led to feel responsible for discriminating as to the conditions. His responsibility ceases when he has placed a filling, crown, or plate that passes inspection. His professional pride has not even been aroused, if indeed awakened. Might not those eligible to operate for the day be called one by one to diagnosticate the cases presenting, and thus show their competence of discrimination first, then to proceed with pride and stimulated observation to justify their positions?

Do surgical aspects present to the student any part of his practical training? Is it of much avail for the class to file by the operating-chair and be told, this is a case of epulis, carcinoma, pyorrhœa alveolaris, or some other surgical aspect of oral lesion?

Should not he be asked to diagnosticate the case, and then, or subsequently, to justify himself, or be set right?

The etiology of dental lesions is worthy of more extended consideration than it receives in college instruction. Investigation by the student is not encouraged, nor indeed are time and facilities at his disposal for such purpose.

The period of preparation is all too short to leave room to combat inconsistencies or uncertainties presented for memorizing that the examination may be passed.

One grave objection to making charges for infirmary operations is the low estimate it insensibly cultivates in the minds of the student of the money value of the services he is rendering. If this system must continue, and the abolishing of all charges is not possible, then the amount is certainly unwise for the student to learn.

Perhaps the actual cost of materials used in a college infirmary may be a legitimate charge, but to put a profit thereon seems undignified and unprofessional, as well as illiberal, and, if known to the student, is inimical to the cultivation of the moral tone that urges him to value his services as skilled professional efforts and not the work of an artisan to be charged for as so much material and time consumed, in which consideration he rapidly drifts away from an appreciation of the skill, anxiety, forbearance, judgment, moral and magnetic force, that all go to make each operation possess a value of its own, distinct from any rule of charges.

This one point is prominently before us, and when so-called extraordinary fees (extraordinary to those whose competence does not reach perhaps even what should be ordinary degree) are known to be asked for what might fairly be termed extraordinary operations, surprise and criticism are freely expressed. Who but the individual operator knows the cost of performing personal service?

*Discrimination* as developed and directed by a proper, broad preparation includes diagnosis, prognosis, and treatment (therapeusis and operation), and marks itself as essential to successful practice. The direction during preparation to the development of discrimination, like manual training, can probably never be overdone.

Discrimination is necessary first to effectively apply effort by reason of a proper predicate being taken from which to determine the course to pursue if

*Preventive;* then advice as to aseptic conditions and personal conduct of the patient in the care of the mouth and teeth and general health, more particularly with regard to children, seems to about cover the ground. The purpose of prevention would well be served by popular instruction through society publication as to which diseased conditions of common occurrence are amenable to treatment, with a concise but clear statement of their salient symptoms; added to which might be a reference to such operations and appliances as the society endorsed. Thus would the public be brought to some adequate appreciation of dental operations, and be prepared to resist needless mutilation and to seek treatment instead of neglecting it.

If discrimination points to reparative effort, then, as long as present circumstances and conditions prevail, the real work of the dentist is called for. Treatment of diseased, and complete or partial removal of effete, useless, irreparable, or redundant hard or soft tissue presents its special indications for medicinal or surgical interference, whether stimulation, depletion, compression, distention, irritation, coagulation, escharotization, solution, injection, aspiration, interruption, cessation, incision, excision, amputation, or extraction.[1]

*Reproduction* may be induced through these applications of preparation, and may become an intermediate stage between treatment and substitution. Reparation includes substitution of lost tissue by filling of mechanical or chemical character (the highest expressions of these efforts, especially concerning defective tooth-structure, appear to be oxyphosphate filling with porcelain or metal wearing surfaces, gold to a limited extent only, crowning inside of collars, immovable and movable bridges with and without porcelain gums, and carved continuous gum-plates). Other efforts would embrace sponge-grafting, the implantation of teeth, flexible and resistant supply of deficient functioning tissue (*i.e.*, artificial vela), and artistic restoration of deficient appearances. The apostle of these last-named efforts is well known in this society.

*Association* brings the detail of these several opportunities for beneficent employment within the possibility of comparison, discussion, and adjustment of antagonistic complexions, and opens the opportunity to promulgation of discoveries, real or assumed, so that side-lights of mental action may be directed upon the presented matter, and its usefulness be extended and intensified. So much depends upon the application of facts, theories, methods, and appliances, that presentment to general consideration and discussion is of vital import to even the originator of them.

Dissension has been permitted too long in associations. The effort is often to vanquish the presenter of new matter as a polem-

---

[1] This is intended to indicate the need for a better understanding of the *principles* of medicinal action, rather than empiric use of specific substances, topically or constitutionally; for instance, *depletion* may be by catharsis, capillary phlebotomy, diaphoresis, emesis, diuresis, vesication, or reduction of heart-action by the specific action of drugs inhaled, per gastrum or hypodermically. *Stimulation* may be by irritation, friction, fomentation, or augmentation of heart-action through specific drugs. *Tonization* may be by food as ordinarily understood, and food such as pharmacy presents to us, also by cleanliness, clothing (especially in such localities as the knees, armpits, abdomen, and chest), and by exercise.

ical contest, and the proper purpose of association lost sight of,—*i.e.*, intensification of facts, elision of redundant aspects of a question, correction of misinterpretations, and specific statement of the status of opinion of the association on the questions dealt with, fearlessly presented and published.

Mere prominence of an individual because of his reception as an essayist before a society is an unworthy object for associations to foster. The majority, if held responsible for endorsement of views presented and accepted, would be chary of ill-considered decision.

Prepared discussion can best establish this status of opinion and that which appeals to a majority of assembled minds it is fair to believe worthy of credence, acceptance, and experiment, from which would arise further elaboration of theory and fact onward and upward to an ever-advancing goal for ambition to reach towards.

Associations might properly announce periodically certain theories as held, and invite *pro* and *con* discussion of them from general sources, embracing as fully as possible all presentments of theories, facts, methods, materials, and appliances offered up to the time of the call for papers and prepared discussion to meet the issue.

This could be based on an invitation to present positions and propositions, and suggestions, and in addition on what may be gleaned from society reports and journal articles.

The result of this called discussion could be expected to permit an arrangement as nearly complete as may be of all bases for practice before the profession, and something like a competent and regulated effort at compiling a system of American dentistry be possible.

The assembling of matter in the transactions of a congress of all associations would gather periodically everything presented anywhere and correlate it in such serial connected order as to render investigation and reference easier as well as broader, and such selected matter as secured the endorsement of the congress would have a value and power unknown to most of the methods we now employ.

The elision of the individuality as supreme in our gatherings is strongly indicated when we consider the antagonism in methods for nearly identical operations.

Investigation being directed to unsettled issues, the concentration of mental effort, not being wasted in considering ground already proved, would secure positive results speedily attained.

*Publication* naturally closely follows association deliberations, and makes them effective on a more extended basis. The record thus spread abroad furnishes sign-boards for other mentalities to move by, and cumulative treading of these paths marks them plainer and plainer, and they thus are made easier to follow, and become highways of recognized truth from certain starting-points to definite destinations. They may require repaving from time to time, but this is discovered and directed by those traversing the road, and the corrections easily applied at slight expense of effort because of the definiteness and circumscribing of the road made possible from the common intent and effort used in its construction.

Publication, as at present before us, appears to present three points for improvement:

*First.* Editing by a board of three to seven practising dentists, perhaps under one supervising chief, instead of being at the discretion of a single censor, in some cases not even a dentist.

*Second.* The proof-reading of but a very few journals is above reproach.

*Third.* Liberal management that would promptly meet the press of matter occasion brings by adequate increase of pages for its accommodation. This would present related matter together for comparison and collateral consideration. Papers are often written to meet issues which time changes the aspect of, and were the publication of them delayed for even one month their relevance to their original incitement would be weakened and perhaps lost.

This is more particularly true of such subjects as are considered in this paper and those embracing organization, ways and means, propositions for experiment, and such as relate to system rather than to scientific facts and methods and appliances, although here the matter of establishing priority alone would seem to urge immediate publication.

In this connection Dr. Ottolengui[1] has presented a consistently arranged scheme, which is well worth serious consideration. To its recommendations is here added that the society reports be published as they occur, in the, let us say, INTERNATIONAL DENTAL JOURNAL, under an editing board, and that a semi-annual or yearly retrospect be issued which shall include those first and second best supported presentments of facts, methods, suggestions, and appliances as have appeared during the preceding period, under some such heading as "endorsed selections." In which retrospect might be a department for contributions especially written for it, presum-

---

[1] *Dental Review*, January, 1890, "Looking Forward."

ably of superior merit. All of which, receiving the specific endorsement of such an organ, could be reasonably expected to be of greatly extended benefit, and in which journal it would be a real honor to appear.

Would not such a stimulus advance the quality of our published matter materially?

*A few of the Points a Congress might consider.*—Does amalgam meet any proper professional object not better fulfilled by oxy-phosphate cement provided with porcelain or metal-wearing surfaces?

Is pulp capping commendable?

Is there any adequate reason for the use of rubber, celluloid, or other non-conducting materials for base plates, or for permanent use in more or less extended contact with mucous membrane?

Have we no method for constructing a denture competent to displace non-conducting materials?

Is pyorrhœa alveolaris curable?

Is this disease of constitutional origin in whole or part?

Is the etiology of dental caries possible of definite solution.

Is delay of operation ever indicated in clearly diagnosticated caries or necrosis of bone?

This is intended to call attention to some of the matter that might be considered at the International Dental Congress suggested for 1893, by arranging a plan having definite announced intentions.

It is hoped the discusssion will deal with this matter fully, in order that organized thought may be directed to an adequate comprehensive plan for the operations of the Congress, from which alone properly useful results can follow. We cannot afford to waste time in politics; we need to work and with a definite purpose.

*Some Suggestions for the Action and Endorsement or Condemnation of this Society, being mainly a Synopsis of this Paper.*—1. Does private pupilage tend to develop better and more capable operators?

2. Pupilage permits selection of material, which indicates that certain qualities must be inborn to make a truly professional man possible.

3. May not clinical instruction be extended and perfected, to the student's and school's profit?

4. Chemistry does not receive the attention in college courses to which it seems justly entitled.

5. Diagnosis seems to require more attention, if the college course may be measured by the lack of this ability apparent on investigation.

6. May not a better knowledge of surgery be possible to impart to the student?

7. Charges for infirmary operations have a pernicious influence on the mind of the student, if responsible for no other evil. It seems proper every effort should be made to elevate the moral status of the student.

8. Proper discrimination does not seem to be a common result of dental education.

9. Prevention is the first service of the dentist.

10. Repair brings out his executive ability, and illustrates the breadth of his training.

11. The principles of medicinal action rather than empiric use of remedies seems worthy of more attention.

12. Surgery embraces not only removal of diseased tissue, but also its reproduction as well as substitution to supply its loss.

13. Intercommunication in societies brings out the facts useful to the presenter of subjects equally with the listener.

14. Discussion does not mean dissension.

15. Does this society approve of a resolution requiring each essayist to present his paper practically completed to the Executive Committee at least a week previous to the time set for reading it, from which three or more copies shall be made and handed to members competent and willing to prepare adequate discussion upon them?

16. Will not a biennial congress, not necessarily international, conducted on an organized comprehensive plan, work our advancement?

17. Editing of scientific subjects should be at the hands of several persons practically engaged in the special branch.

18. Is it impracticable to publish within three months or less the full text of all society proceedings accepted, to be regularly contributed?

19. Is a national examining board for all colleges impracticable?

20. Is not the establishing of one or more purely dental surgery hospital wards essential and worthy of immediate consummation?

21. Cannot a dental club be organized, equipped with sufficient rooms and apparatus and library, for clinical, experimental, and literary work, social intercourse, and the entertainment of guests?

22. May not meetings be devoted to papers and discussions, by referring all business and transient matters to a permanent business committee?

23. Outside of this committee, do not a permanent chairman and secretary fulfil all useful need for officers?

Many of these observations and suggestions have been elaborated in various ways in other quarters, under the same patronymic as the authorship of this paper,—to wit, INTERNATIONAL DENTAL JOURNAL, August, 1889; *Dental Cosmos*, November, 1889; *Ohio Journal*, February, 1890; *Dental Review*, March, 1890; *Transactions of the American Dental Association*, 1889, and in a paper before the Brooklyn Dental Society in February last but not yet published.

It is hoped that the present occasion may be productive of some definite results. Of extended influence they will be if discussed to an issue and that issue published.

